# Response: No evidence for association between polygenic risk for multiple sclerosis and MRI phenotypes in approximately 30,000 healthy adult UK Biobank participants

**DOI:** 10.1177/13524585221079044

**Published:** 2022-03-12

**Authors:** C Louk de Mol, Rinze F Neuteboom, Philip R Jansen, Tonya White

**Affiliations:** 1 Department of Neurology, MS Center ErasMS, Erasmus University Medical Center Rotterdam, Rotterdam, The Netherlands; 2 The Generation R Study Group, Erasmus University Medical Center Rotterdam, Rotterdam, The Netherlands; 3 Department of Complex Trait Genetics, Center for Neurogenomics and Cognitive Research, Amsterdam Neuroscience, Amsterdam University Medical Centers, Amsterdam, The Netherlands; 4 Department of Human Genetics, Amsterdam University Medical Centers, Amsterdam, The Netherlands; 5 Department of Child and Adolescent Psychiatry, Erasmus University Medical Center Rotterdam, Rotterdam, The Netherlands; 6 Department of Radiology and Nuclear Medicine, Erasmus University Medical Center Rotterdam, Rotterdam, The Netherlands

Jacobs et al.^1^ published a study investigating the relationship between polygenic risk for multiple sclerosis (MS) and white matter (WM) alterations in adults from the UK Biobank (UKB) study, with a large sample size of ~30,000 adults. They reported no association between polygenic risk for MS and fractional anisotropy (FA) measures in several WM tracts in the brain after correcting for multiple testing. These results are in contrast with our earlier studies, where we describe a significant association between polygenic risk scores for MS and FA in children from the general population.^2,3^

The findings from Jacobs et al.^1^ are in line with other studies investigating the relationship between genetic MS risk and WM integrity in adults.^4,5^ These earlier studies report similar non-significant associations between MS polygenic risk and FA. Due to the substantial larger sample size, the study by Jacobs et al.^1^ provides more robust evidence against the presence of subclinical magnetic resonance imaging (MRI) brain abnormalities in adults with a high polygenic burden for MS.

An obvious difference between our work and the recent study by Jacobs et al.^1^ is the age of the study population.^2,3^ Both the UKB and the Rotterdam Study involved recruitment of adults older than 40 years of age, whereas our sample included children of 9–11 years. Studies dating back to the 1967 landmark study of Yakolev and Lecours highlight that the neurodevelopment of WM continues throughout childhood and into early-to-middle adulthood.^6^ Within a neurodevelopmental framework, there are multiple explanations for our findings which are not mutually exclusive with the findings of Jacobs et al.^1^ For example, accelerated WM maturation associated with the MS polygenic risk, without an influence in the endpoint in adult WM development.

By including children at a young age in our earlier studies, we were able to investigate possible WM alterations in participants at high polygenic risk of MS before a possible diagnosis of MS later in life.^2,3^ Because of the high median age of the participants in the UKB study, a proportion of the participants was already diagnosed with MS and excluded (around 1 in 240 participants). In addition, in this cohort, brain WM FA is largely influenced by age-related atrophy. Altogether, these factors could explain the differences between study results. Still the possibility remains that children, at risk of being diagnosed with MS later in life, have radiological alterations early in life, a hypothesis that is not possible to validate in the study by Jacobs et al.^1^

In summary, we agree with the observation by Jacobs et al.^1^ that there is little evidence for microstructural MRI alterations in older adults with no diagnosis of MS. However, we believe that their study is neither a replication, nor that their findings and ours are mutually exclusive. We argue that the answer lies in the question of development and that studies investigating possible microstructural brain alterations during development and prior to the “main” risk window of MS will be of great value to understand the pathophysiology behind MS.
